# An Electrical Resistance Diagnostic for Conductivity Monitoring in Laser Powder Bed Fusion

**DOI:** 10.3390/s24020523

**Published:** 2024-01-15

**Authors:** Saptarshi Mukherjee, Edward Benavidez, Michael Crumb, Nicholas P. Calta

**Affiliations:** Lawrence Livermore National Laboratory, Livermore, CA 94550, USA; benavidez7@llnl.gov (E.B.); crumb1@llnl.gov (M.C.); calta1@llnl.gov (N.P.C.)

**Keywords:** electrical resistance measurement, poisson equations, finite difference methods, electrodes, electrical conductivity, laser powder bed fusion, additive manufacturing, nondestructive evaluation

## Abstract

With the growing interest in metal additive manufacturing using laser powder bed fusion (LPBF), there is a need for advanced in-situ nondestructive evaluation (NDE) methods that can dynamically monitor manufacturing process-related variations, that can be used as a feedback mechanism to further improve the manufacturing process, leading to parts with improved microstructural properties and mechanical properties. Current NDE techniques either lack sensitivity beyond build layer, are costly or time-consuming, or are not compatible for in-situ integration. In this research, we develop an electrical resistance diagnostic for in-situ monitoring of powder fused regions during laser powder bed fusion printing. The technique relies on injecting current into the build plate and detecting voltage differences from conductive variations during printing using a simple, cheap four-point electrode array directly connected to the build plate. A computational model will be utilized to determine sensitivities of the approach, and preliminary experiments will be performed during the printing process to test the overall approach.

## 1. Introduction

There is growing interest in additive manufacturing (AM) using laser powder bed fusion (LPBF) due to its capability to generate a wide breadth of geometries and feature sizes for printable geometries such as cellular lattice structures, which exhibit impressive mechanical and thermal performance characteristics, that are particularly useful for next-generation lightweight and multi-functional engineering applications [1,2,3]. The parts produced by LPBF exhibit significantly improved microstructures compared to wrought material produced from conventional manufacturing, but are known to possess defects in the form of pores, cracks, creep or embrittlement [4,5,6,7,8]. As the complexity of these advanced structures increases and approaches manufacturing tolerances and limits, they also present unique challenges for investigating such defects. Not only do these defects degrade performance, but they are also difficult to detect and localize for quality assurance and control using traditional inspection approaches [9,10,11]. These issues motivate advanced diagnostics that can not only detect defects in the printed parts, but provide critical information in-situ during the manufacturing process [12,13,14]. In-situ experimental measurements can provide unparalleled insights into the physics of the process, complementing and validating both high-fidelity modeling approaches [15] and conclusions drawn from ex-situ analysis [16].

Several X-ray synchrotron diagnostics for high-speed imaging of the LPBF process have been developed in the past five years [17,18,19,20]. These techniques aim to understand the sub-surface material behavior and dynamics during laser-induced melting such as quantifying pore formation mechanisms [21], spatter ejection mechanisms [22], melt pool and vapor depression morphologies [23,24], liquid flow in the melt-pool [25], and other physical phenomena during the LPBF process [26]. The melting pattern directly influences the forming results such as changes between conduction to transition to keyhole mode and different spattering velocities [27]. These efforts require costly, time-consuming, and high-volume projection data to provide high-fidelity information about the process. Thus, there is an outstanding need to connect observations made with synchrotron experiments [28] or time-consuming ex-situ inspection approaches to cheap, scalable process monitoring diagnostics that do not rely on synchrotron infrastructure to draw conclusions about the behavior of the LPBF process. Full topical reviews on in-situ diagnostics for LPBF are given in [29,30]. Among them, a few complementary diagnostics involve pyrometric readings to monitor laser melting [31], optical tomography for defect detection [32], acoustics emission measurements for identifying porosities [33], laser-based ultrasound to detect surface and sub-surface defects in laser melt lines [34], thermionic emission for resolving laser-metal dynamics [35] as well as machine learning and sensor data fusion approaches to reliably predict the onset of pore formation [36,37]. These techniques mostly probe the surrounding regions of the melt pool, rather than the integrity of the entire printed part. On the other hand, electromagnetic monitoring (e.g., electrical resistance variations from lack of fusion or temperature fluctuations) can provide crucial information about the integrity of the printed part, such as mechanical discontinuities and defects.

For such a need, a current injection-based method, such as electrical resistance measurements, could be used to detect discontinuities in resistances in metals [38]. The electrical resistance technique initially gained popularity in geophysics in the 1920s to obtain maps of the Earth’s sub-surface resistance [39,40]. The technique has since been widely developed in a broad range of applications, including biomedical imaging applications [41], detecting damage or strain states in construction materials [42,43]. Here, we develop a real-time, electrical resistance experimental diagnostic as a nondestructive evaluation (NDE) method for analyzing prints in an LPBF technique.

**Figure 1:**
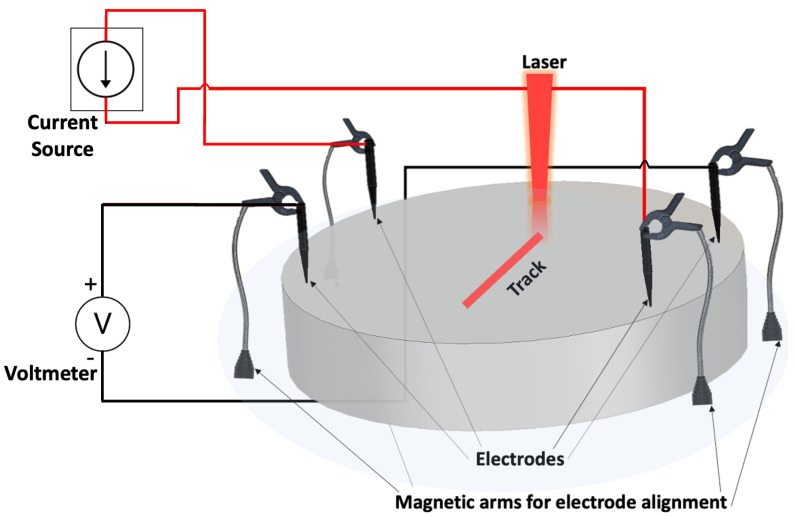
Proposed electrical resistance diagnostic during a LPBF print process. Electrodes are used to inject currents in the printed area. Changes in measured electric voltage from resistance variations along the current flow direction during printing is used to monitor the printing process.

There are several challenges of adapting this technology towards AM metal part inspection [44]. While electrical resistance tomography (ERT) approaches exist that reconstruct the resistance distribution of a conductive target based on boundary electric voltage measurements, they rely on numerous electrode pairs and multiple current injection patterns followed by computationally intensive tomographic reconstruction algorithms to reconstruct resistance values [45,46]. This limits the technique’s ability to be minimally invasive and perform real-time detection during a time-sensitive printing process. To address this, we employ a minimally-invasive simple four-electrode approach (two for current injection, two for voltage measurement) to study the effect of single and multiple LPBF-tracks and prints on the diagnostic measurements (Figure 1). Additionally, the surface area of the current injection electrodes needs to be large enough to allow high current density, while the small surface area of the voltage measurement electrodes needs to be small enough to avoid ’averaging’ several equipotentials. Here, we utilize electrodes with tapered tips that can provide high current over a uniform area (5 A), along with the capability to measure small voltage fluctuations (up to 0.1 μV). Finally, the prior literature on resistance measurements has relied on invasive techniques such as directly soldering electrodes onto the part [47] or additional connection support built onto the part [48,49] to reduce contact impedance mismatch and provide efficient electrical contact. Here, in an attempt to be minimally invasive, we utilize magnetic arms that help maintain a strong electrical connection along with high degree of mechanical flexibility. The thermionic emission approach is an electrical monitoring technique that directly measures electrons from the metal surface during LPBF and can resolve laser-material interaction dynamics [35]. However, this approach may be sensitive to static currents and grounding issues. Our developed electrical resistance approach directly injects current into the build plate, which primarily dictates the current pathways. This method is thus less sensitive to external and background effects such as grounding or static currents. The developed electrical resistance diagnostic offers an easy, cost-effective technique to detect electrical conductivity changes due to process variations such as lack of fusion and temperature fluctuations. This technique is complementary to other existing diagnostics.

A numerical model is developed to identify the key physical mechanisms of current interaction with the laser printed hatches, determine the expected voltage measurement values at the AM relevant spatial scales and optimize our experimental setup. Experimental measurements on LPBF 3-D printed parts and single layer hatches showed sensitivity to printed hatch size, electrode positions and injection patterns. Experiments demonstrated measurable voltage changes due to changes in material resistance as a function of build size and detection of up to a single-trace print. It is important to note that even if the experimental measurements can not provide enough spatial representation to reconstruct an electrical resistance map and identify specific defect locations, they can detect off-normal resistance variations, which could be adequate for process monitoring. To the best of our knowledge, these are the first-ever electrical resistance (DC) experiments on LPBF-printed hatch geometries still attached to the build plate. This work successfully demonstrates the sensitivity of the electrical resistance diagnostic towards resistance variations between single LPBF tracks and unmodified baseplate material and lays the foundation for real-time, in-situ deployment of an electrical measurement system for resistance monitoring at and below the build layer during the manufacturing process.

## 2. Simulation Model

A 2-D simulation model is developed to model the electrostatic field distribution in the region of interest. The Poisson’s equation is utilized to compute the scalar electric potential equation in a region Ω as:

(1) ∇·(σ∇ϕ)=j,inΩ

where σ,ϕ and j are electrical conductivity, electric voltage and current distribution, respectively. The above equation is numerically solved using a finite difference approach [50]. The finite difference representation in 2-D (x, y) of the above equation is given by

(2) $$\left(\partial_{x}+\partial_{y}\right)\left(\sigma \left(x,y\right)\left(\partial_{x}+\partial_{y}\right)\phi \left(x,y\right)\right)=j\left(x,y\right).$$

The final expression for solving ϕ is given by

(3) $$\begin{array}{c} \phi =K^{-1}\;J, \end{array}$$

J is the current distribution matrix and K is the stiffness matrix represented in a sparse diagonalized form to increase the computational speed of the matrix inversion.

(4) $$\begin{array}{c} K=\left(\left(\partial_{x}^{2}+\partial_{y}^{2}\right)\sigma \left(x,y\right)+\partial_{x}\sigma \left(x,y\right)\partial_{y}+\partial_{y}\sigma \left(x,y\right)\partial_{x}\right). \end{array}$$

The following boundary condition is used to truncate the computational domain:

(5) $$\alpha \sigma \frac{\partial \phi }{\partial n}+\beta \phi =0,\;\mathrm{on}\;\tau$$

$\frac{\partial \phi }{\partial n}$ denotes the normal component of the gradient of ϕ on the surface τ, and α,β are functions defined on τ, that are not simultaneously zero.

The simulation model is used to inject electric current and compute electric voltage distribution in a 316L stainless steel build plate with a LPBF printed stainless steel hatch area. The build plate of 25 mm × 25 mm with a conductivity of $1\times 10^{6}$ S/m is considered. A hatch of length 10 mm, depth of 100 μm and widths varying from 250 μm to 1.75 mm is introduced. The electrical conductivity of the hatch is varied from $0.8\times 10^{6}$ S/m to $0.2\times 10^{6}$ S/m, based on temperature-dependent values obtained from the literature [51]. The overall area is discretized into $25\times 10^{4}$ unit cells, with a unit cell size of 50 μm. A pair of electrodes denoted by ‘+’ are used to inject current (of opposite polarities injected on opposite sides of the hatch) across the hatch and a pair of electrodes denoted by ‘o’ are used for voltage measurements. While more current injection and voltage measurement electrodes can be utilized to obtain a higher spatial resolution, here we use two pairs of electrodes to be minimally invasive and reduce practical limitations associated with the manufacturing process. It is important to note that the simulation model does not model the melt-pool or complex physical transformations associated with the LPBF process, as we wanted to primarily understand the effect of the electrical currents with the hatch geometries. Modeling the melt-pool and corresponding physical transitions would lead to additional voltage perturbations and current pathway variations, which are not captured in this model.

**Figure 2:**
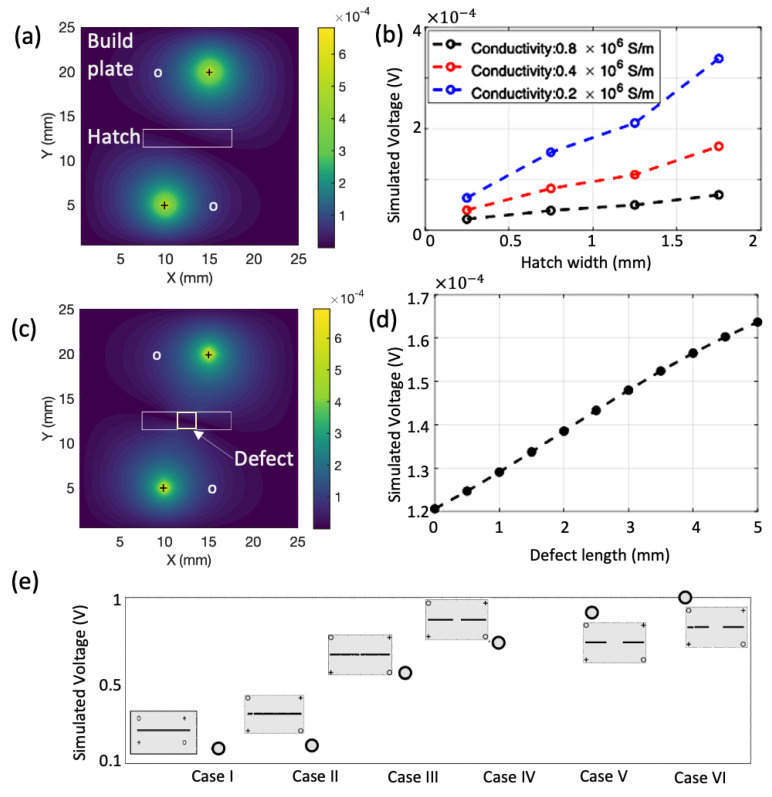
Simulation model results: (**a**) Electric voltage distribution in a build plate with a printed hatch, (**b**) Effect of conductivity and hatch width on the electric voltage, (**c**) Electric voltage distribution in a build plate with a printed hatch with a defect, (**d**) Effect of the defect length on the electric voltage, (**e**) Electric voltage for different defect configurations (indicated in the insets). Case I represents a build plate with no defect, Case II represents an off-centered defect, Cases III–V represent a centered defect with increasing lengths, Case VI represents a configuration with two defects (one centered and one off-centered). ‘+’ denote current electrode and ‘o’ denote voltage measurement electrode.

Figure 2a shows the absolute value of the electrical voltage distribution in a hatch region. The presence of the hatch region perturbs the electrical current pathway, resulting in a change in the voltage distribution, in comparison to the region without a hatch. The position of the electrodes are such that it is located far enough from the current electrodes to measure the presence of conductive anomalies without being dominated by the input source currents. The effect of the current injection patterns will be discussed in detail in the experimental section. Figure 2b shows the effect of the computed voltage as a function of the electrical conductivity and the width of the printed hatch region. A decrease in the electrical conductivity of the hatch leads to an increase in resistance across the hatch, resulting in a higher measured voltage. Similarly, for a fixed conductivity value, increasing the width of the hatch increases the hatch cross sectional area. This leads to a higher overall resistance across the hatch, resulting in a higher computed voltage.

Figure 2c shows the absolute value of the electrical voltage distribution in a hatch region with a defect area at the center. The presence of the defect can be a result of different process dynamics, as discussed in the introduction before. A defect will present either as a lack of fusion (un-welded region) or a void/porosity leading to increased resistance. Here, we consider the un-welded defect case. The presence of the defect region perturbs the electrical current pathway, resulting in more voltage flowing across the region, in comparison to a hatch region without a defect (healthy case). Figure 2b shows the effect of the measured voltage as a function of the length of the defect, ranging from no-defect to a defect with a length of 5 mm. Presence of the defect results in a lower resistance change in comparison to the healthy case, resulting in a higher voltage. Finally, Figure 2e shows the simulated voltage for different defect configurations indicated by cases I through VI (indicated in the insets). Firstly, the presence of defects (Cases II–VI) result in higher voltage, in comparison to without defects (Case I) as explained before. Secondly, it is seen that as the size of the defect increases (Cases III–VI), the voltage increases. Thirdly, an offset in the position (off-centered) of the defect (Case II) results in a reduced voltage due to less current interaction with the defect region in comparison to a centered defect that is located directly on the current injection pathway. Finally, the presence of multiple defects (Case VI) increases the overall voltage due to greater perturbation of the electric currents by the defects.

The simulation model not only helps in understanding the physics of current interaction with the laser printed hatches, but also helps us in determining the expected voltage measurement values at the AM relevant spatial scales and, thus, helps optimize our experimental results. The simulation model is also used to compare and better understand our experimental results, as will be discussed in the next section.

## 3. Experimental Results

### 3.1. Experiments on 3-D Printed Part: Effect of Current Injection Patterns

**Figure 3:**
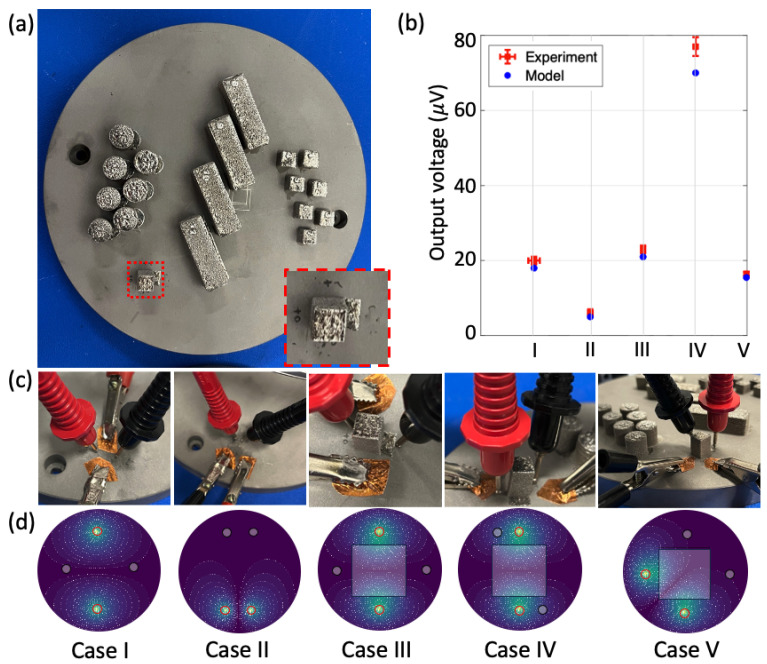
Experimental results on LPBF 3-D printed part: (**a**) SS build plate with several printed parts using LPBF process, with the isolated cuboid highlighted in red and zoomed-in on an inset as our selected printed part for the electrical measurements, (**b**) experimental results for different cases with varying current injection and measurement configuration (Cases I and II refer to measurements with no part, while cases III–V refer to measurements with the printed part), (**c**) experimental setup with five different current injection-voltage measurement configuration, (**d**) simulated electric voltage distribution for all the cases help understand the physics and experimental results.

Preliminary electrical measurements were conducted on a build plate with printed parts to determine the measurable voltage levels and effect of different current injection patterns and measurement modes due to the printed part. These measurements were used as a platform to understand the voltage challenges in being able to measure voltage changes due to a full 3-D print and optimize the experimental setup for enhanced sensitivity and detection capability towards single-layer hatches. A 316L Stainless Steel build plate with several printed parts using LPBF was used for these measurements (Figure 3a). The highlighted red region that corresponds to an isolated cuboid print of dimensions 10 mm × 10 mm × 20 mm in the build plate is utilized for this study. An identical clean build plate with no prints is used for comparison.

While conventional ERT techniques rely on an array of electrode pairs for high-fidelity, here, we we rely on a four electrode measurement strategy with electrode positions and injection patterns optimized to primarily interact with and exhibit high sensitivity to the build plate along with the printed part. Different current injection patterns and measurement modes were tested experimentally to determine sensitivity to a printed part as well as current injection paths. Five different cases are considered, two involving a plate without prints and the remaining three on the plate with the print (Figure 3c,d), as shown below:

I Opposite injection, orthogonal opposite measurement with no part.
II Adjacent injection, opposite adjacent measurement with no part.
III Opposite injection, orthogonal opposite measurement with a printed part.
IV Opposite injection, opposite diagonal measurement with a printed part.
V Adjacent injection, opposite adjacent measurement with a printed part.

**Figure 4:**
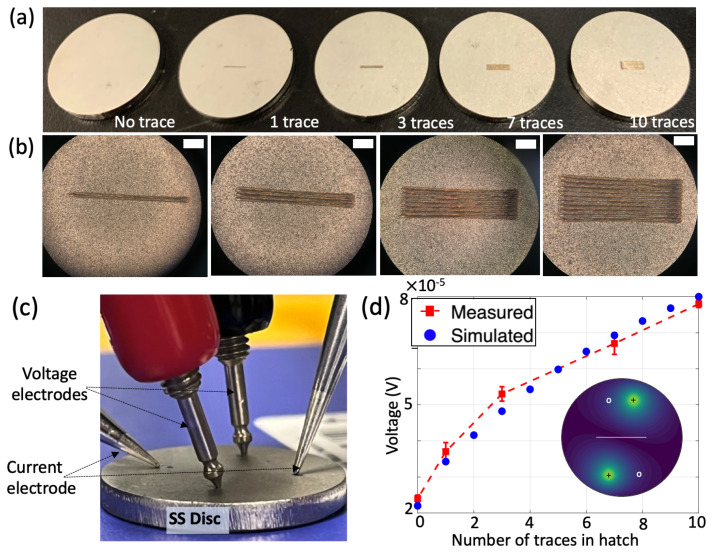
Experimental results on LPBF printed single layer hatches: (**a**) five different SS discs of diameter 1 inch with laser printed hatches of length 4 mm and traces varying from 1 to 10 used for experiments, (**b**) microscopic images (optical zoom: 12×) of the four hatch samples showing the exact width of the hatches (scale: 1 mm), (**c**) experimental system with high-precision magnetic arm-based four electrode system to reliably place the electrodes (close-up picture of the voltage and current electrodes in contact with the disc), (**d**) experimental results for the printed hatches show detection of a single trace hatch and monotonic increase in voltage with increasing traces in hatch, and well-matched with simulation results (inset shows simulated electric voltage distribution in the disc with the current injection-measurement configuration to help understand the physics, ‘+’ denote current electrode and ‘o’ denote voltage measurement electrode).

A Keithley 2230G-30-1 DC power, Keithley Instruments, Cleveland, OH, USA supply was used as the differential current source (current magnitude: 5 A), and an Agilent 34411A $6\frac{1}{2}$ digit digital multimeter, Agilent Technologies, Santa Clara, CA, USA was used for the voltage measurements (Figure 4a). Mechanical pressure was used to contact the voltage measurement electrodes to the plate while the current injection electrodes were directly connected using copper tapes. The measured voltage for the five cases are shown in Figure 3b. A maximum voltage of 78 μV was observed, with a maximum variation of 10 μV. The simulation model was used to compute electric voltage distribution for similar experimental configurations and understand the effect of current injection patterns and voltage measurement locations (Figure 3d). For a similar current and voltage electrode arrangement, the clean plate configuration shows lower voltage than the plate with a printed part (Case I vs. Case III). This is because the presence of the printed part perturbs the electrical current pathway, resulting in a higher voltage change, in comparison to the plate without the printed part. This is significant, as our measurement system shows measurable sensitivity to the printed part. Case IV shows higher sensitivity than the other electrode configurations. This is because the currents are evenly distributed along the entire surface area of the plate. Moreover, the voltage measurement locations are such that they are far enough from the current electrodes to measure the presence of conductive anomalies without being dominated by the input source currents (similarly observed in the simulation section). Case II shows lower sensitivity than the other electrode configurations, as the voltage measurement locations are far away from the the current injection locations. As seen from the simulated voltage distribution, the currents are less distributed along the surface area of the plate and there is minimal contribution of the injected current at the voltage measurement locations. In Case V, the currents are well distributed in the plate surface area. However, since the voltage measurements are quite far from the current injection locations, there is low contribution of the injected current at the voltage measurement locations, thus exhibiting lower sensitivity than Cases III, IV. Cases I and III have better voltage distribution along the entire surface area of the plate than cases II and V and thus exhibit better sensitivity. However, the location of the voltage measurement electrodes for cases I and III are closer to voltage null regions associated with the current injection (as seen from the voltage distribution). As a result, they exhibit lower sensitivity than Case IV. It is important to mention that all X, Y and Z dimensions of the printed part affect the voltage measurements. An increase in the dimensions of the printed part would increase the overall resistance across the part, resulting in a higher computed voltage, as seen in Figure 2b. Finally, simulation results mimicking similar experimental configurations show a close match with the experimental results. Moreover, as indicated above, the simulation model helps better understand the physics and explain the measurement results. Based on this experimental analysis and the different current injection and measurement strategies, the opposite injection with opposite diagonal measurement was selected as the desired technique for resistance monitoring from or during the LPBF printing process.

### 3.2. Experimental Results for Single Layer Prints

The above mentioned experimental study also helped better understand and fix practical experimental challenges and enhance voltage sensitivity due to printed hatches. In that study, we used mechanical pressure to contact the voltage measurement electrodes and copper tape to attach the current injection electrodes to the plate. While this ensured a robust electrical contact, the presence of the copper tapes led to high contact impedance mismatch, leading to decay of injected currents. As a result, the measured voltage values are relatively low. While the values were measurable, increased sensitivity is required for interrogating smaller printed parts and for detecting small sub-surface defects in those parts.

Four magnetic-based metal arms (Kotto Inc., Torrance, CA, USA) are used to connect the electrodes to the build plate with strong mechanical pressure that helps maintain a strong, reliable electrical connection, leading to lower contact impedance losses. The magnetic arms are placed in a steel weighted base that provides a high degree of mechanical flexibility to conform to small geometries, which can be easily translated to a complex manufacturing system with limited space for connection. Further, we connect the electrodes to tapered copper tips, which can provide strong electrical connection and allow high current over a uniform area (5 A). The high-precision tapered copper tips (IGREAT ET Tip Series) are plated with iron, nickel and chromium. They are typically used for soldering purposes and are repurposed for this application. These modifications make the experimental four-point probe system system minimally invasive with optimized electrode current injection with the capability to measure small voltage fluctuations (up to 0.1 μV), without complex circuitry that can be easily translated for in-situ integration.

### 3.3. Effect of Number of Traces of Printed Hatches

The sensitivity of the above electrical resistance diagnostic towards single-layer hatches printed by a laser-based system are studied. Five different coupons, which are stainless steel discs with a diameter of 1 inch were used as the base material. A laser was used to create four single-layer hatches with a length of 4 mm and a number of traces increased from one to ten (one, three, seven, and ten, respectively). The print parameters used to create these hatches are as follows: laser power of 300 W, scan speed of 500 mm/s, laser beam diameter (d4sigma) of 120 μm, hatch spacing of 120 μm and a scan length of 4 mm. The five discs, one without any hatch and the remaining four with hatches of increasing width, are shown in Figure 4b. Microscopic images of the four hatch samples show that the width of the hatch varies from 200 μm for the one trace-sample to 2 mm for the ten trace-sample.

Figure 4c shows a close-up picture of the voltage and current electrodes in contact with the disc, using the magnetic arms and high-precision tips. As mentioned before, this ensures strong electrical connection at the metal boundaries. The opposite injection, opposite diagonal measurement configuration (corresponding to case IV in Figure 3d) was used as the current injection pattern and measurement mode for these measurements, as optimized in the previous section. The experimental results are shown in Figure 4d. The key finding of the measurements is that the effect of a single trace hatch was detected with a measurable voltage of 15 μV greater than a disc with no hatch printed. An increase in the width of the hatch increases the hatch cross sectional area, leading to a higher overall resistance across the hatch, resulting in a higher measured voltage. Thus, the measured voltage increases with increasing resistance due to increasing traces in hatch, with the 10-trace hatch providing an 80 μV voltage. The optimization of the experimental setup and the injection pattern leads to stable measurements, with a maximum variation of 2.5 μV. Finally, simulation results mimicking similar experimental configurations show a close match with the experimental results. The simulated electric voltage distribution (inset of Figure 4d) shows that the voltage distribution is well distributed along the area of the disc and it is perturbed due to the presence of the hatch region. These results demonstrate that a single trace hatch can be easily detected with the experimental system and opens up the possibility of detecting defects within a single trace as well as in-situ conductive monitoring during the LPBF process. While this work uses ex-situ measurements, future work would focus on using this NDE technique for in-situ measurements during LPBF. A potential challenge for in-situ transition is that the electrodes would interfere directly with the recoater and powder spread during LPBF. We hypothesize that the voltage measurements would not be significantly affected due to the small cross sectional contact area from the tapered electrode tips. To further tackle this issue, we will explore the feasibility of placing the electrodes either at the bottom or sides of the build plate and thus eliminate the interference with the recoater and powder, as part of future work. It is worth mentioning that while this work focuses on conductivity changes at the surface that could also be captured by existing diagnostics such as high-speed camera and photodetector sensing, contrary to these existing diagnostics, the developed technique can also be used to detect sub-surface defects. Sub-surface defects during the LPBF process would reduce the material bulk conductivity, thereby impacting the measured potential. This will be studied as part of our future work.

## 4. Conclusions

An electrical resistance experimental diagnostic as an NDE method for analyzing prints in an LPBF-AM technique is discussed. A numerical model is developed to understand the physics of current interaction with the laser-printed hatches, determine the expected voltage measurement values at the AM relevant spatial scales and help optimize the experimental setup. Experiments on LPBF 3-D printed parts illustrated sensitivity to the electrode positions and injection patterns and demonstrated the technique’s ability to detect parts attached to a build plate. Measurements on single layer prints (single and multi-trace hatches) demonstrate the feasibility of this approach and are capable of measuring single trace hatches with a measurable voltage difference of 15 μV. This work successfully demonstrates the sensitivity of the electrical resistance diagnostic towards resistance variations from LPBF tracks and lays the foundation for real-time, in-situ deployment of an electrical measurement system for resistance monitoring at and below the build layer during the manufacturing process. 

## Data Availability

Data are contained within the article.
